# Novel mutations in the ferritin-L iron-responsive element that only mildly impair IRP binding cause hereditary hyperferritinaemia cataract syndrome

**DOI:** 10.1186/1750-1172-8-30

**Published:** 2013-02-19

**Authors:** Sara Luscieti, Gabriele Tolle, Jessica Aranda, Carmen Benet Campos, Frank Risse, Érica Morán, Martina U Muckenthaler, Mayka Sánchez

**Affiliations:** 1 Institute of Predictive and Personalized Medicine of Cancer (IMPPC), Ctra. de Can Ruti, Camí de les Escoles s/n, 08916, Badalona, Barcelona, Spain; 2 Molecular Medicine Partnership Unit (MMPU) EMBL, University of Heidelberg, Heidelberg, Germany; 3 Department of Pediatric Oncology, Hematology, and Immunology, University Hospital of Heidelberg, Heidelberg, Germany; 4 Servicio de hematología y hemoterapia, Hospital Arnau de Vilanova, Valencia, Spain; 5 Praxis für Hämatologie- Onkologie Rhein Ahr, Remagen, Germany

**Keywords:** Serum ferritin, Iron metabolism, IRP/IRE regulatory system, Bilateral cataracts

## Abstract

**Background:**

Hereditary Hyperferritinaemia Cataract Syndrome (HHCS) is a rare autosomal dominant disease characterized by increased serum ferritin levels and early onset of bilateral cataract. The disease is caused by mutations in the Iron-Responsive Element (IRE) located in the 5^′^ untranslated region of L-Ferritin (FTL) mRNA, which post-transcriptionally regulates ferritin expression.

**Methods:**

We describe two families presenting high serum ferritin levels and juvenile cataract with novel mutations in the L-ferritin IRE. The mutations were further characterized by *in vitro* functional studies.

**Results:**

We have identified two novel mutations in the IRE of L-Ferritin causing HHCS: the Badalona +36C > U and the Heidelberg +52 G > C mutation. Both mutations conferred reduced binding affinity on recombinant Iron Regulatory Proteins (IPRs) in EMSA experiments. Interestingly, the Badalona +36C > U mutation was found not only in heterozygosity, as expected for an autosomal dominant disease, but also in the homozygous state in some affected subjects. Additionally we report an update of all mutations identified so far to cause HHCS.

**Conclusions:**

The Badalona +36C > U and Heidelberg +52 G > C mutations within the L-ferritin IRE only mildly alter the binding capacity of the Iron Regulatory Proteins but are still causative for the disease.

## Background

Ferritin is the protein responsible for the storage and intracellular distribution of iron [1]. It is composed of 24 subunits of two types named H- and L-Ferritin, encoded by two different genes. The H subunit generates ferroxidase activity to incorporate iron into the protein shell and the L subunit facilitates iron-core formation. The synthesis of these two proteins is controlled post-transcriptionally by the Iron Regulatory Proteins (IRPs) that bind to the Iron Responsive Element (IRE), a conserved hairpin-like motif, located in the 5^′^ untranslated region (UTR) of ferritin mRNAs [2]. The binding of the IRPs to IREs occurs under iron-deficient conditions and results in translational repression of both ferritins. Mutations in the IRE of Ferritin L (FTL) mRNA cause reduced IRP binding with concomitant up-regulation of FTL synthesis in the Hereditary Hyperferritinaemia Cataract Syndrome (HHCS). HHCS (ORPHA163, OMIM # 600886) was first described in 1995 as an autosomal dominant disease characterized by a combination of high serum ferritin levels with congenital bilateral nuclear cataract and the absence of iron overload [3-6]. Differential diagnosis with hereditary hemochromatosis, a genetic iron-overload disease, is achieved by genetic analysis and biochemical measures of serum iron and transferrin saturation indices, which are not increased in HHCS. In this work we describe two families of Spanish and German origin with HHCS caused by novel mutations in the FTL IRE. Unexpectedly for an autosomal dominant disease, one of the mutations was detected in the homozygous state in some affected members of the Spanish family. *In vitro* studies indicate a minor disturbance of the IRP-IRE binding by these mutations.

## Methods

### Patients

#### Pedigree 1

Proband (III:2, Figure 1A and Table 1) is a 54 year old woman of Spanish origin presenting a 10-year history of hyperferritinaemia with no sign of iron overload. Serum iron, transferrin saturation and liver functional tests were normal. The patient presents no evidence of hepatitis, cirrhosis, diabetes, inflammatory diseases, metabolic syndrome or neoplasia and genetic testing for HFE hereditary hemochromatosis was negative. She has suffered from bilateral cataracts since she was 18 years old and underwent surgery at the age of 39. The proband has one sister (III:4) and a cousin (III:9) presenting similar clinical features with hyperferritinaemia and juvenile bilateral cataract (Table 1). No evidence of elevated serum ferritin was found in four other sisters of the proband. A deceased maternal uncle (II:4) had suffered from hyperferritinaemia and cataract reported as an adult. Notice that the proband’s parents (II:1 and II:2) were first cousins; the mother died from acute myeloid leukemia and the father from senile dementia and he had suffered from cataracts which required surgical correction in adult age. The proband’s mother was never diagnosed with cataracts but she suffered from severe myopia. The proband’s daughter (IV:1), aged 27, shows no cataracts, but she has moderately elevated serum ferritin levels above 200 ng/ml and low transferrin saturation (to be considered with a concomitant hypermenorrhea) (Table 1). The two sons (IV:2 and IV:3) of the other affected sister also have moderately elevated serum ferritin levels and subject IV:2 also shows early signs of cataracts (Table 1).

**Figure 1:**
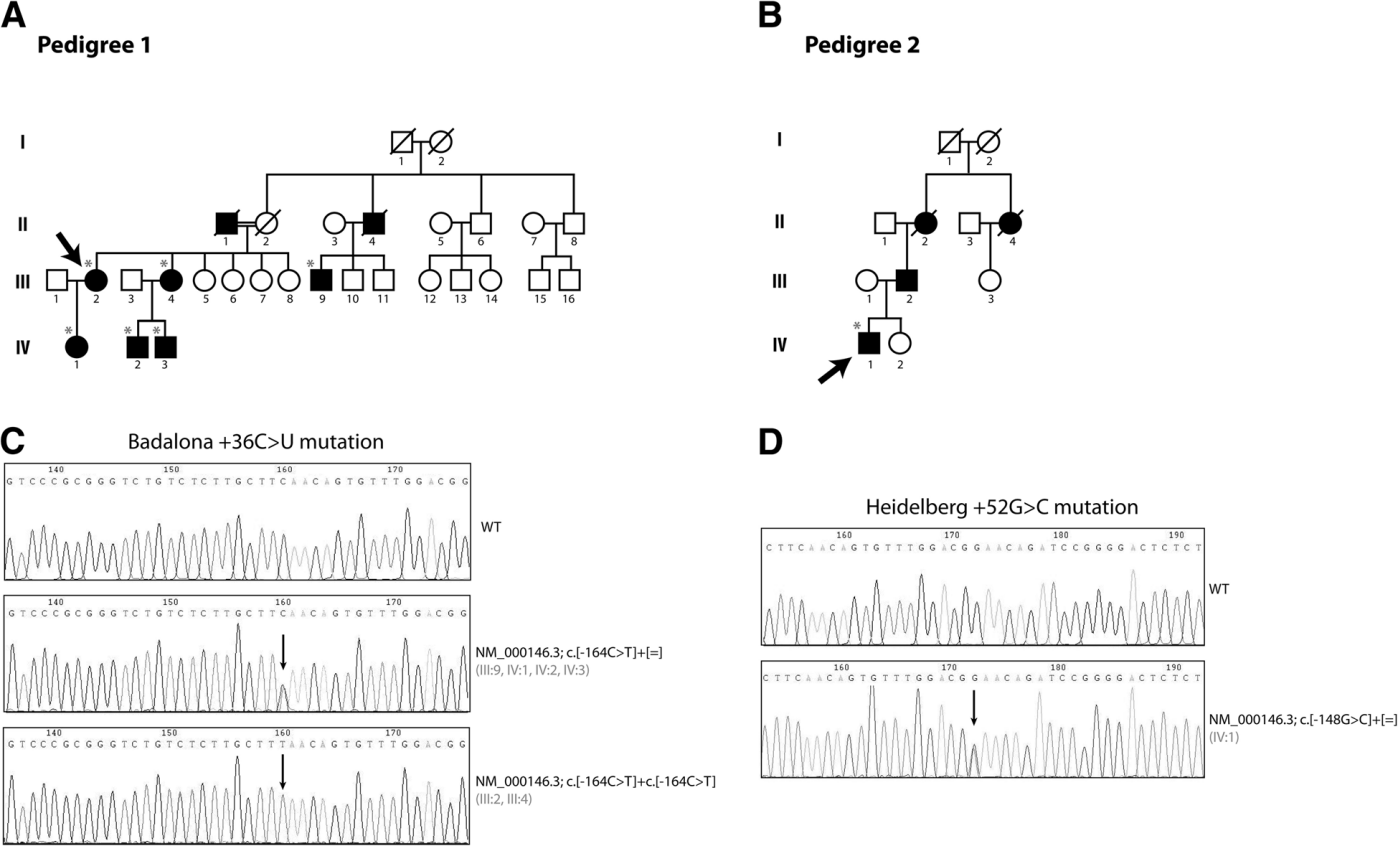
**(A, B) Pedigree trees of the two families with HHCS studied.** Squares indicate males, circles females and rhombus subjects of unknown sex. Filled symbols indicate affected members and barred symbols indicate deceased subjects. Asterisks indicate subjects characterized at the molecular level. (**C**, **D**) Chromatograms of partial FTL IRE sequences where mutations are located. Arrows indicate the nucleotide position that is altered by the pathological mutation. WT denotes wild type sequence according to reference sequence [NCBI:NM_000146.3]. Traditional and conventional HGVS nomenclatures are shown.

**Table 1:** Biochemical and clinical parameters of the affected members of the two pedigrees studied

	**Pedigree 1**						**Pedigree 2**		
**Characteristic**	**III:2 (proband)**	**III:4**	**III:9**	**IV:1**	**IV:2**	**IV:3**	**IV:1 (proband)**	**III:2**	**Normal values**
Age at diagnosis (years)	54	53	48	27	27	25	19	40	
Sex	F	F	M	F	M	M	M	M	
Hb (g/dl)	13.2	13.1	15.3	13.4	14.3	14.7	15.6	15	♂ 13,8–18 ♀ 12,1–15,1
MCV (fl)	92.3	96	94.1	91.3	85.1	83.4	80.9	80,6	79–99
CRP (mg/L)	<5	-	4	<5	<5	-	4	-	5–10
Serum iron (μg/dl)	74	56	79	37	124	243	127	111	37–170
TIBC (μg/dl)	361	-	342	372	327	424	-	-	250–450
Serum ferritin (ng/ml)	719	534	952	239	303	447	1290	1260	♂ 12–300 ♀ 12–200
Transferrin (mg/dl)	252	-	-	260	229	296	291	300	200–360
Transferrin saturation (%)	20	-	23.1	10	38	57	31	25	20–55
ALT (mU/ml)	28	17	35	21	38	14	-	-	14–36
AST (mU/ml)	35	20	40	20	30	30	27	33	9–52
Cataract diagnosis (years)	20	17	20	-	27	-	16	16	
Cataract surgery (years)	39	-	-	-	-	-	-	-	
HFE gene mutation	H63D+/−, S65C−/−, C282Y−/−	H63D−/−, S65C−/−, C282Y−/−	H63D+/+, S65C−/−, C282Y−/−	H63D−/−, S65C−/−, C282Y−/−	H63D−/−, S65C+/−, C282Y−/−	H63D−/−, S65C−/−, C282Y−/−	H63D−/−, S65C−/−, C282Y−/−	-	
Mutation IRE L-ferritin	c.[−164C > T] + c.[−164C > T]	c.[−164C > T] + c.[−164C > T]	c.[−164C > T] + [=]	c.[−164C > T] + [=]	c.[−164C > T] + [=]	c.[−164C > T] + [=]	c.[−148 G > C] + [=]	c.[−148 G > C] + [=]	
	Badalona +36C > U	Badalona +36C > U	Badalona +36C > U	Badalona +36C > U	Badalona +36C > U	Badalona +36C > T	Heidelberg +52 G > C	Heidelberg +52 G > C	

Reference values are indicated in the last column. The following abbreviations were used: Hb, hemoglobin; MCV, mean corpuscular volume; CRP, C-reactive protein; ALT, alanine aminotransferase; AST, apartate aminotransferase. HFE gene mutations refer only to C282Y, H63D and S65C mutations.

#### Pedigree 2

The proband (IV:1, Figure 1B and Table 1) is a 19 year old man originating from Germany, who was referred to the medical doctor due to fatigue and difficulties in concentrating. He showed high serum ferritin levels with normal serum iron and transferrin saturation. He presented with cataracts since the age of 16 and has not undergone surgery. He does not present any other clinical signs. No evidence for acute or chronic inflammation was detected and his liver functional tests and abdominal morphology at echography medical inspection were normal. The test for Hereditary Hemochromatosis type 1 (HFE) was negative and Hereditary Hemochromatosis type 4 (ferroportin disease) was excluded, with no evidence of pathological mutations in the SLC40A1 gene. The father (III:2) presents with similar biochemical findings showing elevated serum ferritin and clinical symptoms with cataracts detected also at the age of 16 (Table 1). The paternal grandmother (II:2) and a great-aunt (II:4) had also suffered from cataracts (Figure 1B).

Written informed consent for genetic analyses was obtained from the probands and relatives of the two families according to the guidelines of the institution and the study protocol conforms to the ethical guidelines of the 2002 Helsinki Declaration.

### PCR amplification and DNA sequencing

Genetic studies were performed with minor differences for the two pedigrees.

Genomic DNA was extracted from peripheral blood using the FlexiGene DNA kit or QIAamp DNA Blood Mini kit (Qiagen) according to manufacturer’s instructions. PCR amplification of exon 1 of L-ferritin was performed with 50 ng of genomic DNA using primers reported in Additional file 1: Table S1. For pedigree 1 the cycling conditions were: denaturation at 94°C, annealing ranging from 66 to 60°C, and extension at 72°C, each step for 30 seconds and for 30 cycles while for pedigree 2 were: denaturation at 95°C, annealing at 58°C, and extension at 72°C, step 1 and 2 for 30 seconds, step 3 for 45 seconds and for 38 cycles. The resulting amplification product was verified on a 2% agarose gel. The PCR product was processed to remove excess dNTPs and unincorporated primers: for pedigree 1, 8 μl of PCR product were treated with 10U Exonuclease I and 10U Antarctic Phosphatase (New England Biolabs) at 37°C for 30 min and the reaction was inactivated by heating at 80°C for 15 min. For Pedigree 2, 40 μl of PCR product were cleaned up using the NucleoSpin Gel and PCR Clean-up Kit (Machery-Nagel) according to manufacturer’s instructions. The purified PCR product was sequenced using conventional Sanger method [7] by GATC BIOTECH company (Konstanz, Germany). Sequencing results were analyzed using Mutation Surveyor software (SoftGenetics LLC) or Chromas software (Technelysium Pty Ltd).

### Plasmid construction

Plasmids for generating EMSA probes were constructed based on the I-12.CAT plasmid [8] by replacing H-ferritin IRE with annealed synthetic oligonucleotides corresponding to the sequences of L-ferritin wild type (WT) or the mutated versions: +39ΔC, Badalona +36 C > U, Milano +36 C > G, Heidelberg +52 G > C or Torino +29 C > G; oligonucleotide sequence are reported in Additional file 1: Table S1. DNA templates were linearized with XbaI and used for *in vitro* transcription.

### Electrophoretic mobility shift assay (EMSA)

EMSAs were performed with a new non-radiolabeled method ([9], and manuscript in preparation) using fluorescently labeled probes with Aminoallyl-UTP-ATTO-680 (Jena Bioscience). For direct and competitive EMSAs we include proper controls such as the +39ΔC deletion construct (positive control), the Milano +36 C > G mutation [10], a variation at the same position as the Badalona +36C > U mutation and the Torino +29C > G mutation [11], the counterpart of the Heidelberg +52 G > C change. In competitive EMSAs unlabeled competitors were *in vitro* transcribed using MEGAscript T7 kit (Life Technologies) according to the manufacturer’s instructions. Experimentally for both assays, 100 ng of labeled probe (plus increasing molar excess of unlabeled competitor probes in the competitive EMSAs), were heated 3 min at 95°C and then incubated with 120 ng of recombinant His-tagged IRP1 or IRP2 protein for 15 min at room temperature. Non-specific RNA-protein interactions were displaced by adding 50 μg of sodium heparin for 10 min. Samples were loaded on a 5% native acrylamide gel in 1x TBE buffer. RNA-protein complexes were visualized using Odyssey Infrared Imager (LI-CORE Bioscience).

### RNA folding predictions

RNA folding analysis of IRE motifs was performed using SIREs Web Server (http://ccbg.imppc.org/sires/, [12]) and RNAfold Web Server (http://rna.tbi.univie.ac.at/cgi-bin/RNAfold.cgi, University of Vienna).

### Statistical analysis

Values were compared using Student’s t-test for unpaired data. Differences were defined as statistically significant for P values less than 0.05.

## Results and discussion

### Molecular genetic studies

Sequencing of exon 1 of L-ferritin (FTL) in two families with HHCS revealed the presence of two previously undescribed sequence variations.

In pedigree 1, a C > U change was detected at position +36 of the upper stem of FTL IRE ([NCBI:NM_000146.3]; c.-164C > T, HGSV nomenclature). Interestingly, the mutation was found to be present in the homozygous state in two members of this Spanish family (the proband, III:2 and her sister, III:4), while other affected members carry the change in heterozygosity (Figure 1A and C). To exclude that homozygosity of the mutation may arise from a “drop of allele mechanism” we have sequenced the FTL exon 1 using two independent sets of primers and identical results were obtained (data not shown). Moreover, possible in *trans* deletions were assessed with gene dosage studies by qPCR demonstrating that all studied patients from pedigree 1 carry two copies of the FTL gene (data not shown).

Sequencing analysis in pedigree 2 showed a G > C change at position +52 of the IRE lower stem ([NCBI:NM_000146.3]; c.-148 G > C) in a heterozygous state (Figure 1B and D).

Following the traditional nomenclature for FTL IRE mutations we refer to these mutations as the “Badalona +36C > U” and “Heidelberg +52 G > C” mutations, respectively. The presence of either the Badalona or Heidelberg variation was further validated by PCR-Restriction Fragment Length Polymorphism, as the +36C > U and +52 G > C changes introduce a MseI and a HgaI recognition site, respectively (data not shown). Both changes were absent in 50 control subjects, which rules out their being neutral polymorphisms and supports their causative role for the disease. Additionally, the sequencing of the complete FTL gene (coding region and exon-intron boundaries) revealed no other changes.

### The Badalona +36C > U and the Heidelberg +52 G > C variants show reduced binding of IRP1 and IRP2

**Figure 2:**
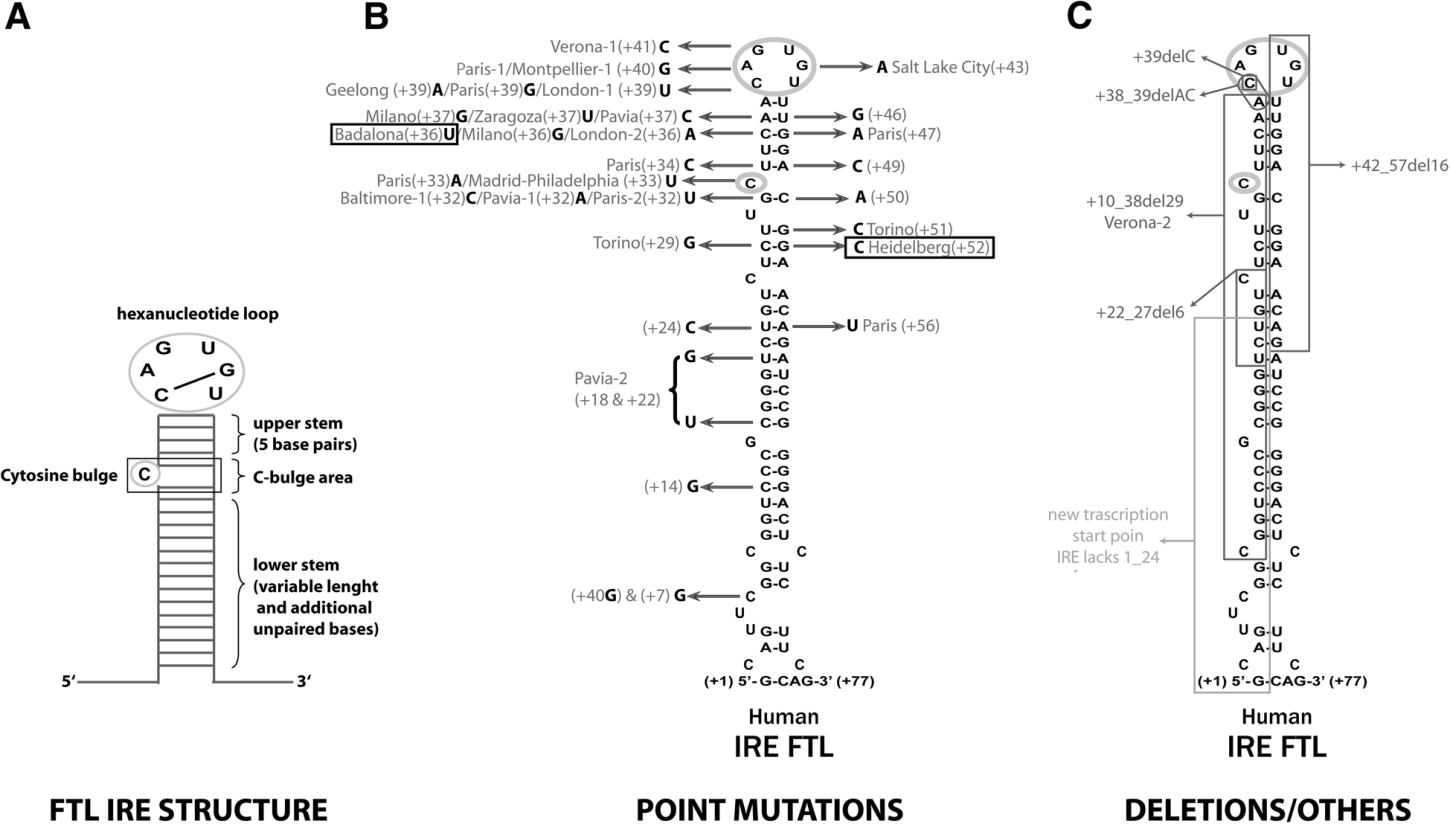
**(A) Schematic representation of the canonical structure of FTL IRE depicting important structural parts: the hexanucleotide apical loop, the cytosine bulge and the upper and lower stems.** (**B**) Picture of the FTL IRE indicating all known causative point mutations in HHCS. The two new mutations described here (Badalona +36C > U and Heidelberg +52 G > C) are boxed. (**C**) Picture of FTL IRE showing the known deletions causing HHCS.

The substitution of guanine by cytosine at position +52 (mutation Heidelberg) is predicted to affect base pairing with the cytosine +29 (Figure 2). However, this is not obvious for the Badalona +36C > U variation because, in theory, the presence of cytosine or uracil at position +36 is not expected to alter base pairing with the guanine at position +47 in the IRE structure (Figure 2), as both C-G or U-G pairing are possible matching pairs within RNA structures. For this mutation we performed folding prediction analysis of the wild-type (WT) and the mutated sequences, using the SIREs and RNAfold Web Servers and the result shows that the Badalona +36C > U substitution increases the folding free energy and opens the structure of the mutated FTL-IRE, when compared to the wild-type IRE (Additional file 1: Figure S1).

**Figure 3:**
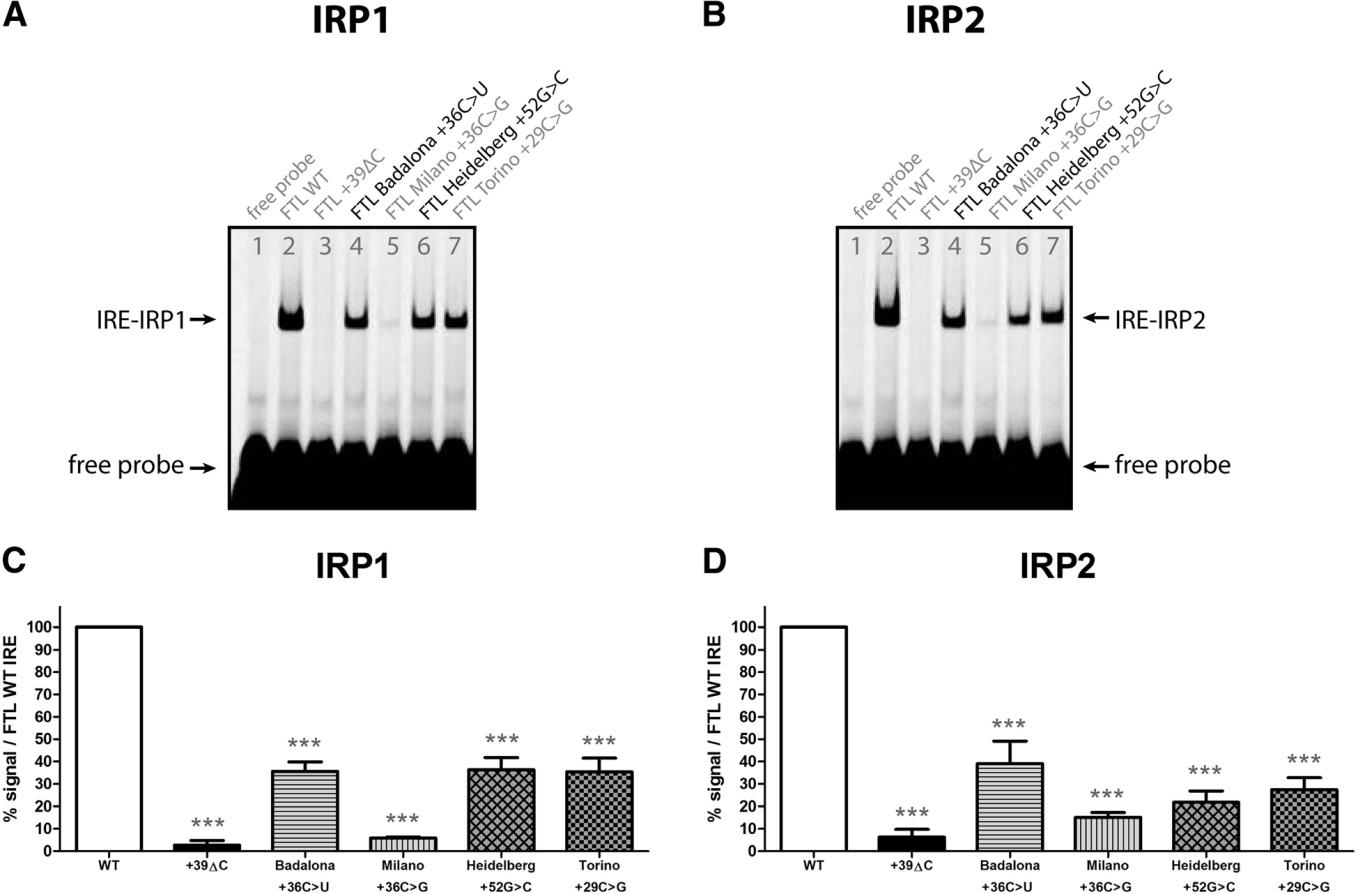
**(A, B) Direct electrophoretic mobility shift assays.** Fluorescent labeled probes corresponding to FTL IRE wild type (lanes 1–2), the mutations +39ΔC (lane 3), Badalona +36C > U (lane 4), Milano +36C > G (lane 5), Heidelberg +52 G > C (lane 6) and Torino +29C > G (lane 7) were incubated with either rIRP1 (panel **A**) or rIRP2 (panel **B**) and the IRP-IRE complex resolved on acrylamide gels. One representative gel is shown. (**C**, **D**) Quantification of the signals in direct EMSAs compared to the wild type FTL IRE signal that was set to 100%. Means ± SD (standard deviation) of at least three independent experiments are shown. *** p < 0.001.

**Figure 4:**
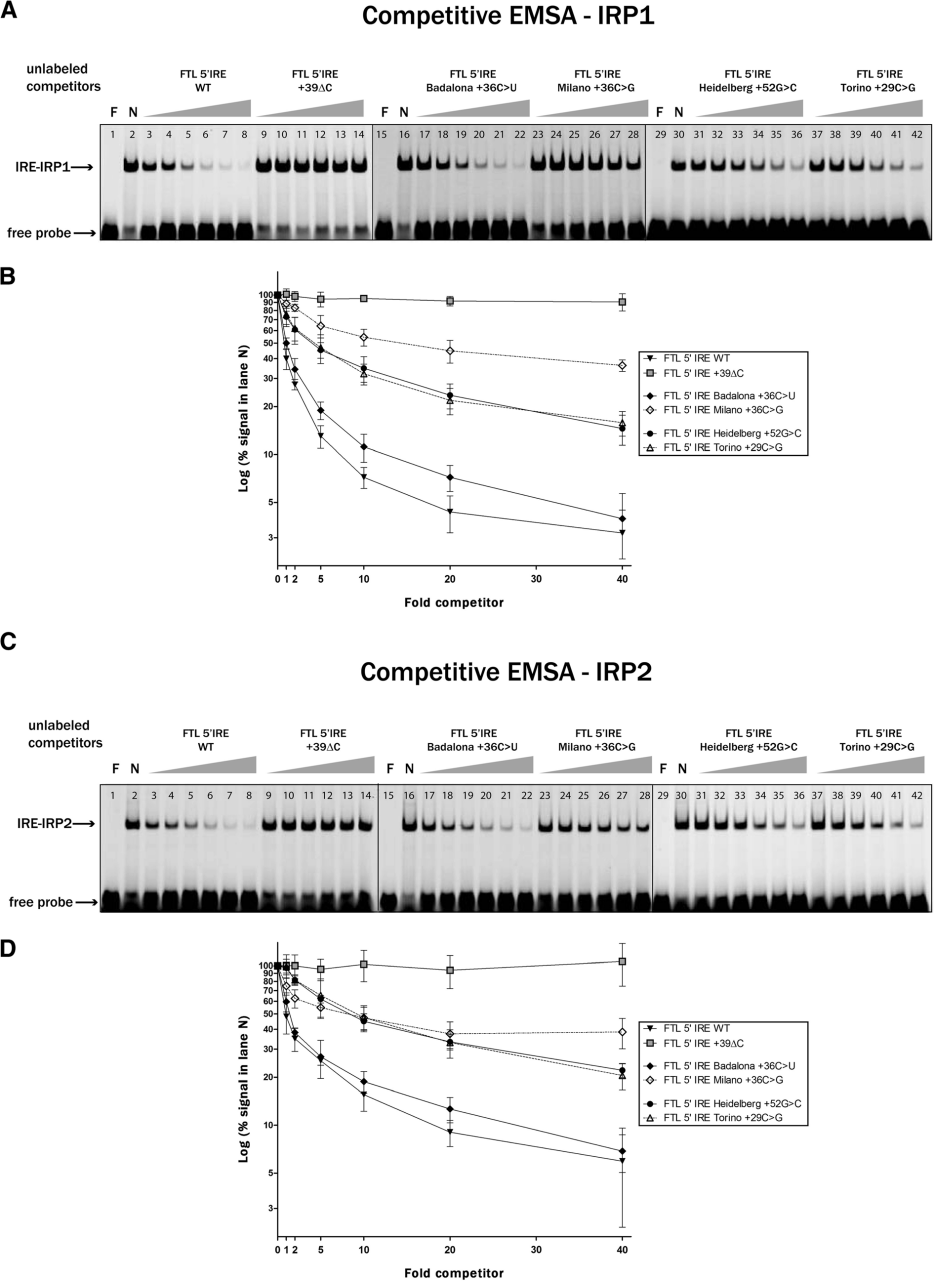
**(A, C) Competitive EMSAs.** Fluorescent labeled FTL wild type probe was incubated with increasing molar excess concentration (1x, 2x, 5x, 10x, 20x and 40x) of unlabeled competitors corresponding to the FTL IRE wild type sequence (lanes 3–8) or the mutants +39ΔC (lanes 9–14), Badalona +36C > U (lanes 17–22), Milano +36C > G (lanes 23–28), Heidelberg +52 G > C (lanes 31–36) or Torino +29C > G (lanes 37–42). Samples were then incubated with either rIRP1 (panel **A**) or rIRP2 (panel **C**) and resolved on acrylamide gels. One representative gel is shown. F indicates free probe and N indicates no competitor added. (**B**, **D**) Quantification of the signals in competitive EMSAs compared to the signal in lane N (taken as 100%) are represented in logarithmic scale. Means ± SD of at least three independent experiments are shown.

For both mutations we next examined the ability of the mutated IRE to bind recombinant IRP1 or IRP2 by electrophoretic mobility shift assays (EMSAs). As expected little IRP binding occurs to the non-functional IRE structure (+39ΔC, Figure 3A and B, lanes 3), which was used as a positive control. Importantly, the Badalona +36C > U and Heidelberg +52 G > C mutations show a reduction of up to 30-40% in the binding to both IRPs (Figure 3A and B, lanes 4 and 6); a similar level of reduction was observed for the Torino +29C > G mutation ([11], Figure 3A and B, lanes 7), while the Milano +36C > G mutation [10] reduces its binding to both IRPs more drastically (Figure 3A and B, lanes 5). Next, we checked the Badalona +36C > U and Heidelberg +52 G > C changes by a more stringent assay, a competitive EMSA. The Badalona +36C > U mutation shows a mild but significant reduction in the efficiency of competition when compared to wild type unlabeled competitor (Figure 4A and C, lanes 17–22 compared to 3–8), while its corresponding control, the Milano +36C > G mutation, is inefficient in displacing the wild type probe; for this mutation a small degree of competition is only appreciable at 20x and 40x molar excess of competitor (Figure 4A and C, lanes 27–28). The Heidelberg +52 G > C mutation also shows a reduced capacity to compete with the FTL WT probe and behaves similarly to its corresponding control, the Torino +29C > G mutation (Figure 4A and C, lanes 31–36 and 37–42). Results obtained for IRP2 were comparable and consistent with previous data showing that IRP1 and IRP2 bind to the L-ferritin IRE with similar affinity (Figures 3 and 4).

### Update on HHCS mutations

Hereditary Hyperferritinaemia Cataract Syndrome (ORPHA163, OMIM #600866) was first described in 1995 by two independent groups in Italy and France [3-6]. Additional file 1: Table S2 summarizes all 37 reported mutations causing HHCS, including the two novel mutations reported here. 31 of these are point mutations and 6 are deletions of different sizes. The majority of the causative mutations are located in the hexanucleotide loop, followed by the C-bulge region, the upper stem and the lower stem of the IRE structure (Figure 2). Other occurrences of inherited unexplained hyperferritinaemia but without cataracts or cataracts diagnosed in adult age have been attributed to mutations in the promoter region, coding region or outside the IRE motif of FTL [10,13-15].

**Figure 5:**
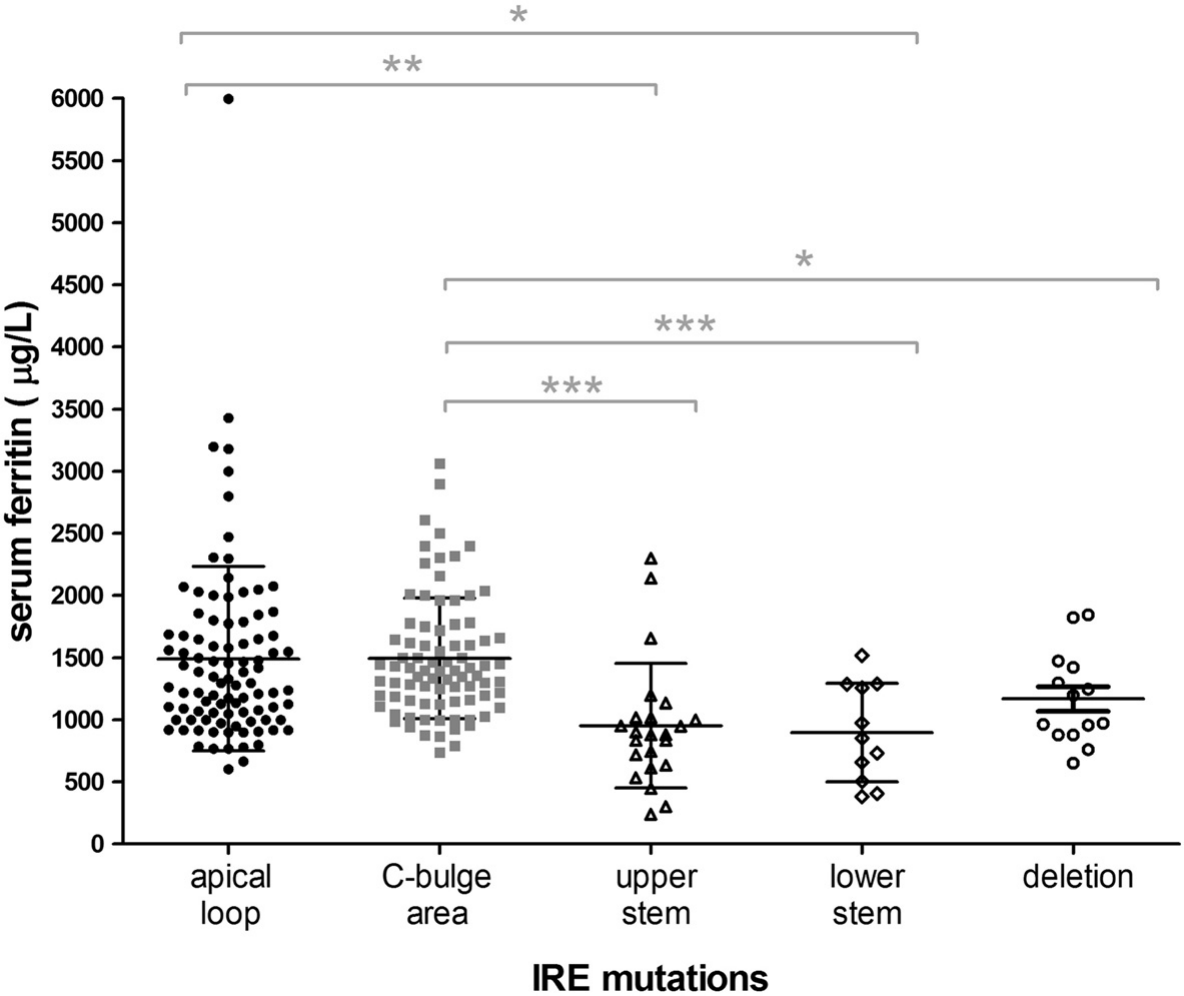
**Phenotype-genotype correlation in all described HHCS patients.** Serum ferritin levels (y-axis) are reported against the position of the IRE mutation in FTL IRE (x-axis). Patients with mutations affecting the apical loop or the C-bulge area present higher serum ferritin levels compared to the ones with mutations in the upper or lower stems. *p < 0.05; **p < 0.01; ***p < 0.001.

Several authors have attempted to correlate the clinical severity of the disease with the position of the IRE mutation [16,17]. An extensive analysis of all described cases in the literature demonstrates that serum ferritin levels correlate with key IRE substructures (Figure 5). Mutations affecting the most important IRE structural elements, such as the hexanucleotide loop or the C-bulge area are detected in patients with more elevated serum ferritin levels compared to those patients with mutations affecting the base pairing of the upper or lower stem of the IRE (Figure 5). Consistently, our cases with mutations in the upper (Badalona mutation) and lower (Heidelberg mutation) IRE stem also show intermediate serum ferritin levels (<1300 ng/ml).

HHCS is inherited as an autosomal dominant trait in all reported families and few cases have been described with *de novo* mutations [18-24]. Homozygous mutations are very unusual in HHCS. Indeed, apart from the case we report here, only one other patient has been reported [25].

The geographical distribution of HHCS patients is worldwide although most cases have been identified in Europe and the USA; most probably due to the localization of specialized laboratories [26]. Global prevalence of the disease has not been clearly defined. In an attempt to screen more than 3000 blood donors and almost 13000 patients with cataract [27] no mutations were detectable in the L-ferritin IRE, suggesting that HHCS is a rare disease. Its prevalence has been estimated to be 1 in 200000 in the Australian population [19].

## Conclusions

In this report we describe two families who have HHCS due to two novel mutations in the L-ferritin IRE. Unexpectedly for an autosomal dominant disease, one of these families carries the mutation in a homozygous state in some affected subjects. Within this family there is a tendency for correlation between the genotype of the subjects and the clinical severity of the disease. However, this correlation is not perfect due to associated factors (age, sex, particular clinical history) that make difficult comparison between subjects. Therefore, we confirm that, as previously reported, a phenotype/genotype correlation in HHCS is difficult to establish due to concomitant pathologies, clinical penetrance and the fact that serum ferritin levels are influenced by sex and age and are subjected to inter and intra-individual variability. By *in vitro* assays we show that these mutations mildly impair IRP-IRE binding; however this minor disturbance is sufficient for biochemical and clinical symptoms to occur in the patients. As previously demonstrated by others we also confirm a tendency for a correlation between the position of the IRE mutation and the ferritin levels in this disease.

Moderate hyperferritinaemia is a common feature found in the adult population and it can be attributed to different factors including metabolic disease, liver dysfunction, neoplasia, infection and inflammation [28]. Some of these cases could be due to the rare genetic disease HHCS. Therefore, proper tests are important for a correct diagnosis for hyperferritinemia and to avoid unnecessary phlebotomy treatment in the case of HHCS.

## Abbreviations

IRP: Iron regulatory protein; IRE: Iron responsive element; UTR: Untranslated region; FTL: Ferritin L; HHCS: Hereditary Hyperferritinaemia cataract syndrome; EMSA: Electrophoretic mobility shift assay; PCR: Polymerase chain reaction; TBE: Tris-borate-EDTA; WT: Wild-type; SD: Standard deviation; NA: Not available; Hb: Hemoglobin; MCV: Mean corpuscolar volume; CRP: C-reactive protein; ALT: Alanine aminotransferase; AST: Apartate aminotransferase.

## Competing interests

The authors declare that they have no competing interests.

## Authors’ contributions

MS is the principal investigator and takes primary responsibility for the paper. CBC and FR recruited the patients. SL, JA, EM and GT performed the laboratory work for this study. MS and MUM co-coordinated the research. SL, MS and MUM wrote the paper. All authors read and approved the final manuscript.

## Supplementary Material

Additional file 1: Table S1 Primer sequences used for genetic diagnosis and for vector construction in EMSAs. **Figure S1.** (A, B) Structure of the IRE motif of the wild type (panel A) or Badalona +36C > U mutation (panel B). (C, D) RNAfold Web Server folding predictions of the FTL IRE wild type (panel C) or the Badalona +36C > U change (panel D). Colored scale indicates the probability of base pairing from 0 (low, blue) to 1 (high, red). **Table S2.** Table summarizing all HHCS mutations described up to now in the literature. The table shows for each mutation, the conventional nomenclature according to HGVS (corresponding to [NCBI:NM_000146.3] reference sequence), the traditional nomenclature, the position in the IRE structure, the number of families and patients described, the patients’ ancestry and the corresponding published report. In bold is indicated the first time a mutation was described; § indicates *de novo* mutations; NA (not available). Click here for file
