# Supplementary material for: Novel mutations in the ferritin-L iron-responsive element that only mildly impair IRP binding cause hereditary hyperferritinaemia cataract syndrome
Source: Orphanet J Rare Dis. 2013 Feb 19;8:30. doi: 10.1186/1750-1172-8-30 (PMC3585816; doi:10.1186/1750-1172-8-30)
Supplement: Additional file 1: Table S1 — Primer sequences used for genetic diagnosis and for vector construction in EMSAs. Figure S1. (A, B) Structure of the IRE motif of the wild type (panel A) or Badalona +36C > U mutation (panel B). (C, D) RNAfold Web Server folding predictions of the FTL IRE wild type (panel C) or the Badalona +36C > U change (panel D). Colored scale indicates the probability of base pairing from 0 (low, blue) to 1 (high, red). Table S2. Table summarizing all HHCS mutations described up to now in the literature. The table shows for each mutation, the conventional nomenclature according to HGVS (corresponding to [NCBI:NM_000146.3] reference sequence), the traditional nomenclature, the position in the IRE structure, the number of families and patients described, the patients’ ancestry and the corresponding published report. In bold is indicated the first time a mutation was described; § indicates de novo mutations; NA (not available). [file 1750-1172-8-30-S1.pdf]

Additional files

Additional table 1. Primer sequences used for genetic diagnosis and for vector construction in EMSAs.

Additional figure 1. (A, B) Structure of the IRE motif of the wild type (panel A) or Badalona +36C>U mutation (panel B). (C, D) RNAfold Web Server folding predictions of the FTL IRE wild type (panel C) or the Badalona +36C>U change (panel D). Colored scale indicates the probability of base pairing from 0 (low, blue) to 1 (high, red).

Additional table 2. Table summarizing all HHCS mutations described up to now in the literature. The table shows for each mutation, the conventional nomenclature according to HGVS (corresponding to [NCBI:NM_000146.3] reference sequence), the traditional nomenclature, the position in the IRE structure, the number of families and patients described, the patients' ancestry and the corresponding published report. In bold is indicated the first time a mutation was described; § indicates *de novo* mutations; NA (not available).

Additional table 1

Primers used for PCR and plasmids construction

Name	Sequence 5' -> 3'	Purpose
hs_FTL_2F	GGGCTGAGACTCCTATGTGC	PCR and sequencing (pedigree 1)
hs_FTL_2R	GCAGCTGGAGGAAATTAGGG	PCR and sequencing (pedigree 1)
hs_FTL_for	GGGCTGAGACTCCTATGTGC	PCR and sequencing (pedigree 2)
hs_FTL_rev	GACTCCGCCCTCTGTTTACC	PCR and sequencing (pedigree 2)
hsFTL-IRE-WT-forward	CGGTCCCGCGGGTCTGTCTCTTGCTTCAACAGTGTTTGGACGGAACAGATCCGGGGACTT	EMSA probe generation
hsFTL-IRE-WT-reverse	CTAGAAGTCCCCGGATCTGTTCCGTCCAAACACTGTTGAAGCAAGAGACAGACCCGCGGGACCGGTAC	EMSA probe generation
hsFTL-IRE-39ΔC-forward	CGGTCCCGCGGGTCTGTCTCTTGCTTCAAAGTGTTTGGACGGAACAGATCCGGGGACTT	EMSA probe generation
hsFTL-IRE-39ΔC-reverse	CTAGAAGTCCCCGGATCTGTTCCGTCCAAACACTTTGAAGCAAGAGACAGACCCGCGGGACCGGTAC	EMSA probe generation
hsFTL-IRE-36C>U-Badalona-forward	CGGTCCCGCGGGTCTGTCTCTTGCTTTAACAGTGTTTGGACGGAACAGATCCGGGGACTT	EMSA probe generation
hsFTL-IRE-36C>U-Badalona-reverse	CTAGAAGTCCCCGGATCTGTTCCGTCCAAACACTGTTAAAGCAAGAGACAGACCCGCGGGACCGGTAC	EMSA probe generation
hsFTL-IRE-36C>G-Milano-forward	CGGTCCCGCGGGTCTGTCTCTTGCTTGAACAGTGTTTGGACGGAACAGATCCGGGGACTT	EMSA probe generation
hsFTL-IRE-36C>G-Milano-reverse	CTAGAAGTCCCCGGATCTGTTCCGTCCAAACACTGTTCAAGCAAGAGACAGACCCGCGGGACCGGTAC	EMSA probe generation
hsFTL-IRE-52G>C-Heidelberg-forward	CGGTCCCGCGGGTCTGTCTCTTGCTTCAACAGTGTTTGGACGCAACAGATCCGGGGACTT	EMSA probe generation
hsFTL-IRE-52G>C-Heidelberg-reverse	CTAGAAGTCCCCGGATCTGTTGCGTCCAAACACTGTTGAAGCAAGAGACAGACCCGCGGGACCGGTAC	EMSA probe generation
hsFTL-IRE-29C>G-Torino-forward	CGGTCCCGCGGGTCTGTCTGTTGCTTCAACAGTGTTTGGACGGAACAGATCCGGGGACTT	EMSA probe generation
hsFTL-IRE-29C>G-Torino-reverse	CTAGAAGTCCCCGGATCTGTTCCGTCCAAACACTGTTGAAGCAACAGACAGACCCGCGGGACCGGTAC	EMSA probe generation

Additional figure 1

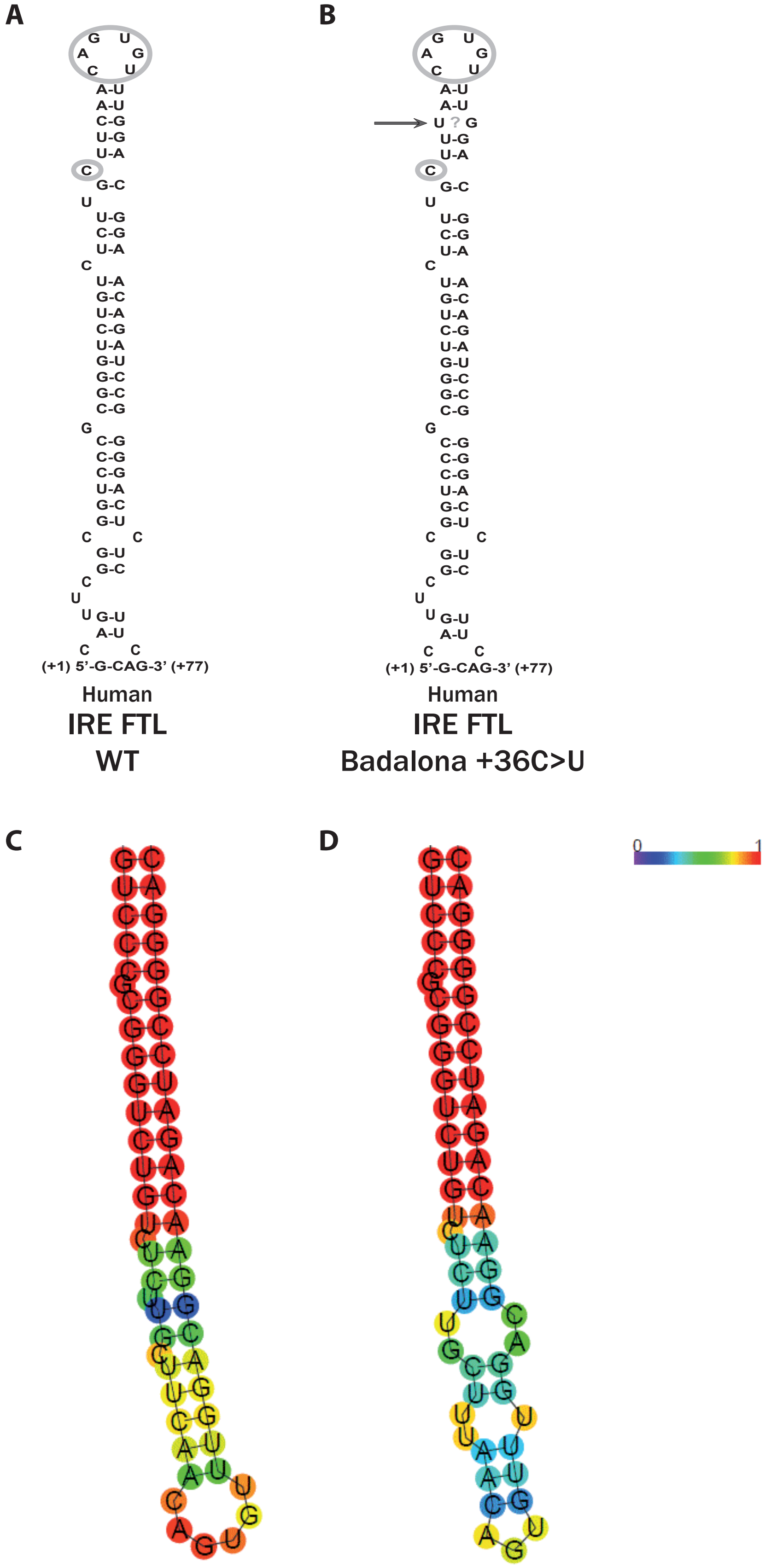

Additional table 2

Summary of HHCS identified mutations (September 2012)

HGVS nomenclature [NCBI:NM_000146.3]	Traditional nomenclature	Mutation position	N°families / patients	Country origin of patients	Reference publication
**c.-193C>G + c.-160A>G**	**+7C>G & +40A>G**	**lower stem + hexanucleotide loop**	**1/1**	**Italy**	**[1]**
**c.-186C>G**	**+14C>G**	**lower stem**	**1/1**	**Italy**	**[2]**
**c.-182c>T + c.-178T>G**	**Pavia-2 +18C>U & +22U>G**	**lower stem**	**1/3**	**Italy**	**[3]**
c.-182c>T + c.-178T>G	Pavia-2 +18C>U & +22U>G	lower stem	NA	NA	[4]
**c.-176T>C**	**+ 24U>C**	**lower stem**	**1/3**	**Switzerland**	**[5]**
**c.-171C>G**	**Torino +29C>G**	**lower stem**	**1/2**	**Italy**	**[6]**
c.-171C>G	Torino +29C>G	lower stem	1/4	Spain	[7]
**c.-168G>A**	**Pavia-1 +32G>A**	**lower stem**	**1/8**	**Italy**	**[3]**
c.-168G>A	Pavia-1 +32G>A	lower stem	1/4	Italy	[8]
c.-168G>A	Pavia-1 +32G>A	lower stem	1/5	Italy	[9]
c.-168G>A	Pavia-1 +32G>A	lower stem	NA	NA	[4]
c.-168G>A	Pavia-1 +32G>A	lower stem	1/7	Netherlands	[10]
c.-168G>A	Pavia-1 +32G>A	lower stem	2/5	France	[11]
c.-168G>A	Pavia-1 +32G>A	lower stem	1/2	Netherlands	[12]
c.-168G>A	Pavia-1 +32G>A	lower stem	1/1	Italy	[13]
c.-168G>A	Pavia-1 +32G>A	lower stem	1/15	India	[14]
c.-168G>A	Pavia-1 +32G>A	lower stem	1/12	Spain	[15]
c.-168G>A	Pavia-1 +32G>A	lower stem	1/17	Germany	[16]
c.-168G>A	Pavia-1 +32G>A	lower stem	1/13	USA (German ancestry)	[17]
**c.-168G>C**	**Baltimore-1 +32G>C**	**lower stem**	**NA**	**Italy**	**[18]**
c.-168G>C	Baltimore-1 +32G>C	lower stem	NA	NA	[4]

c.-168G>C	Baltimore-1 +32G>C	lower stem	1/7	Italy	[19]
c.-168G>C	Baltimore-1 +32G>C	lower stem	1/4	Australia (English ancestry)	[20]
c.-168G>C	Baltimore-1 +32G>C	lower stem	1/10	Netherlands	[10]
c.-168G>C	Baltimore-1 +32G>C	lower stem	1/2	France	[11] §
c.-168G>C	Baltimore-1 +32G>C	lower stem	2/7	UK (1 patient of Thai ancestry)	[21]
c.-168G>C	Baltimore-1 +32G>C	lower stem	1/NA	UK	[22]
c.-168G>C	Baltimore-1 +32G>C	lower stem	1/1	Switzerland	[23]
c.-168G>C	Baltimore-1 +32G>C	lower stem	1/4	Israel (Rumanian ancestry)	[24]
**c.-168G>T**	**Paris-2 or Milano-1 +32G>U**	**lower stem**	**2/8**	**France / Italy**	**[25]**
c.-168G>T	Paris-2 or Milano-1 +32G>U	lower stem	1/1	Australia	[20] [26] §
c.-168G>T	Paris-2 or Milano-1 +32G>U	lower stem	NA	NA	[4]
c.-168G>T	Paris-2 or Milano-1 +32G>U	lower stem	5/>6	France	[11]
c.-168G>T	Paris-2 or Milano-1 +32G>U	lower stem	1/2	UK	[27]
c.-168G>T	Paris-2 or Milano-1 +32G>U	lower stem	1/1	Italy	[13]
c.-168G>T	Paris-2 or Milano-1 +32G>U	lower stem	1/5	UK	[21]
c.-168G>T	Paris-2 or Milano-1 +32G>U	lower stem	1/8	Canada	[28]
c.-168G>T	Paris-2 or Milano-1 +32G>U	lower stem	1/3	Israel	[29]
c.-168G>T	Paris-2 or Milano-1 +32G>U	lower stem	1/4	Germany	[30]
c.-168G>T	Paris-2 or Milano-1 +32G>U	lower stem	1/9	USA (British/German ancestry)	[17]
c.-168G>T	Paris-2 or Milano-1 +32G>U	lower stem	1/1	Spain	[7] §
**c.-167C>A**	**Paris +33C>A**	**C bulge**	**1/1**	**France**	**[31]**
c.-167C>A	Paris +33C>A	C bulge	NA	NA	[4]
c.-167C>A	Paris +33C>A	C bulge	1/1	France	[11]
c.-167C>A	Paris +33C>A	C bulge	1/4	Switzerland	[23]
**c.-167C>T**	**Madrid or Philadelphia +33C>U**	**C bulge**	**1/6**	**Spain**	**[32]**
c.-167C>T	Madrid or Philadelphia +33C>U	C bulge	NA	NA	[4]

c.-167C>T	Madrid or Philadelphia +33C>U	C bulge	1/5	USA	[33]
c.-167C>T	Madrid or Philadelphia +33C>U	C bulge	3/>4	France	[11]
c.-167C>T	Madrid or Philadelphia +33C>U	C bulge	1/1	Italy	[13]
c.-167C>T	Madrid or Philadelphia +33C>U	C bulge	1/6	Spain	[34]
c.-167C>T	Madrid or Philadelphia +33C>U	C bulge	1/4	UK	[21]
c.-167C>T	Madrid or Philadelphia +33C>U	C bulge	1/3	Spain	[35]
c.-167C>T	Madrid or Philadelphia +33C>U	C bulge	1/1	Ireland	[36] §
c.-167C>T	Madrid or Philadelphia +33C>U	C bulge	1/4	Spain	[37]
c.-167C>T	Madrid or Philadelphia +33C>U	C bulge	1/4	Spain	[38]
c.-167C>T	Madrid or Philadelphia +33C>U	C bulge	1/2	Germany	[39]
c.-167C>T	Madrid or Philadelphia +33C>U	C bulge	1/3	Spain	[7]
**c.-166T>C**	**Paris +34U>C**	**upper stem**	**1/3**	**France**	**[11]**
**c.-164C>A**	**London-2 +36C>A**	**upper stem**	**1/9**	**UK**	**[40]**
c.-164C>A	London-2 +36C>A	upper stem	NA	NA	[4]
c.-164C>A	London-2 +36C>A	upper stem	1/1	France	[11]
c.-164C>A	London-2 +36C>A	upper stem	1/17	UK	[21]
**c.-164C>G**	**Milano +36C>G**	**upper stem**	**1/1**	**Italy**	**[13]**
c.-164C>G	Milano +36C>G	upper stem	1/2	Italy	[41]
**c.-164C>T**	**Badalona +36C>U**	**upper stem**	**1/7**	**Spain**	**this study**
**c.-163A>T**	**Zaragoza +37A>U**	**upper stem**	**1/7**	**Spain**	**[35]**
**c.-163A>C**	**Pavia +37A>C**	**upper stem**	**1/1**	**Italy**	**[42]**
c.-163A>C	Pavia +37A>C	upper stem	1/6	Italy	[43]
**c.-163A>G**	**Milano +37A>G**	**upper stem**	**1/1**	**Italy**	**[13]**
**c.-161C>A**	**Geelong +39C>A**	**hexanucleotide loop**	**1/2**	**Australia (Italian ancestry)**	[20] **[26]**
**c.-161C>G**	**Paris +39C>G**	**hexanucleotide loop**	**1/3**	**France**	**[44]**
c.-161C>G	Paris +39C>G	hexanucleotide loop	3/19	Greece	[45]

**c.-161C>T**	**London-1 +39C>U**	**hexanucleotide loop**	**1/2**	**UK**	**[40]**
c.-161C>T	London-1 +39C>U	hexanucleotide loop	1/1	Italy	[46] §
c.-161C>T	London-1 +39C>U	hexanucleotide loop	1/8	Italy	[8]
c.-161C>T	London-1 +39C>U	hexanucleotide loop	NA	NA	[4]
c.-161C>T	London-1 +39C>U	hexanucleotide loop	3/5	France	[11]
c.-161C>T	London-1 +39C>U	hexanucleotide loop	1/1	Italy	[13]
c.-161C>T	London-1 +39C>U	hexanucleotide loop	1/4	UK	[21]
c.-161C>T	London-1 +39C>U	hexanucleotide loop	1/11	Austria	[47]
c.-161C>T	London-1 +39C>U	hexanucleotide loop	1/2	Belgium	[48]
c.-161C>T	London-1 +39C>U	hexanucleotide loop	1/1	Spain	[49] §
c.-161C>T	London-1 +39C>U	hexanucleotide loop	1/NA	Italy	[50]
c.-161C>T	London-1 +39C>U	hexanucleotide loop	1/11	Germany	[39]
c.-161C>T	London-1 +39C>U	hexanucleotide loop	1/3	Spain	[51]
**c.-160A>G**	**Paris-1 or Montpellier-1 +40A>G**	**hexanucleotide loop**	**1/5**	**France**	**[52]**
c.-160A>G	Paris-1 or Montpellier-1 +40A>G	hexanucleotide loop	1/3	France	[53]
c.-160A>G	Paris-1 or Montpellier-1 +40A>G	hexanucleotide loop	1/6	UK	[54]
c.-160A>G	Paris-1 or Montpellier-1 +40A>G	hexanucleotide loop	1/2	UK (French/German ancestry)	[55]
c.-160A>G	Paris-1 or Montpellier-1 +40A>G	hexanucleotide loop	1/8	Italy	[8]
c.-160A>G	Paris-1 or Montpellier-1 +40A>G	hexanucleotide loop	1/6	UK	[56]
c.-160A>G	Paris-1 or Montpellier-1 +40A>G	hexanucleotide loop	1/NA	Spain	[57]
c.-160A>G	Paris-1 or Montpellier-1 +40A>G	hexanucleotide loop	NA	NA	[4]
c.-160A>G	Paris-1 or Montpellier-1 +40A>G	hexanucleotide loop	1/18	Australia (Irish ancestry)	[20] [26]
c.-160A>G	Paris-1 or Montpellier-1 +40A>G	hexanucleotide loop	4/>7	France	[11]
c.-160A>G	Paris-1 or Montpellier-1 +40A>G	hexanucleotide loop	1/1	Italy	[13]
c.-160A>G	Paris-1 or Montpellier-1 +40A>G	hexanucleotide loop	1/6	UK	[21]
c.-160A>G	Paris-1 or Montpellier-1 +40A>G	hexanucleotide loop	2/7	Belgium (1 family of Turkish ancestry)	[48]

c.-160A>G	Paris-1 or Montpellier-1 +40A>G	hexanucleotide loop	1/1	Italy	[58]
c.-160A>G	Paris-1 or Montpellier-1 +40A>G	hexanucleotide loop	1/3	Spain	[59]
c.-160A>G	Paris-1 or Montpellier-1 +40A>G	hexanucleotide loop	1/3	UK	[60]
c.-160A>G	Paris-1 or Montpellier-1 +40A>G	hexanucleotide loop	1/NA	Italy	[50]
c.-160A>G	Paris-1 or Montpellier-1 +40A>G	hexanucleotide loop	1/3	Germany	[61]
c.-160A>G	Paris-1 or Montpellier-1 +40A>G	hexanucleotide loop	1/2	UK	[62]
c.-160A>G	Paris-1 or Montpellier-1 +40A>G	hexanucleotide loop	2/9	Germany	[39]
c.-160A>G	Paris-1 or Montpellier-1 +40A>G	hexanucleotide loop	1/3	Belgium	[63]
**c.-160A>G + c.-159G>C**	**Paris-1 or Montpellier-1 +40A>G & Verona-1 +41G>C**	**hexanucleotide loop**	**1/3**	**Italy**	**[2, 64]**
**c.-159G>C**	**Verona-1 +41G>C**	**hexanucleotide loop**	**1/9**	**Brazil (Spanish ancestry)**	**[65]**
**c-157G>A**	**Salt Lake City +43G>A**	**hexanucleotide loop**	**1/14**	**USA (German ancestry)**	**[66]**
**c.-154T>G**	**+46U>G**	**upper stem**	**1/4**	**Italy**	**[50]**
**c.-153G>A**	**Paris +47G>A**	**upper stem**	**1/3**	**France**	**[11]**
**c.-151A>C**	**+49A>C**	**upper stem**	**1/1**	**Italy**	**[1]**
**c.-150C>A**	**+50C>A**	**lower stem**	**1/11**	**Mexico**	**[67]**
**c.-149G>C**	**Torino +51G>C**	**lower stem**	**1/2**	**Canada**	**[68]**
c.-149G>C	Torino +51G>C	lower stem	1/2	USA	[69]
**c.-148G>C**	**Heidelberg +52G>C**	**lower stem**	**1/4**	**Germany**	**this study**
**c.-144A>T**	**Paris +56A>U**	**lower stem**	**1/1**	**France**	**[42]**
**NG_008152.1; g.4980_5004del25**		**New transcription starting site (resulting IRE lacks nt 1-24)**	**1/12**	**Australia**	**[70]**
**c.-190-162del29**	**Verona-2 +10_38del29**	**eliminating IRE**	**1/6**	**Italy**	**[71]**
**c.-178-173del6**	**+22_27del6**	**eliminating part of lower stem**	**1/2**	**Italy**	**[72]**
c.-178-173del6	+22_27del6	eliminating part of lower stem	1/1	Italy	[13]
**c.-162-161delAC**	**+38_39del AC**	**eliminating part hexanucleotide loop**	**NA**	**NA**	**[4]**
c.-162-161delAC	+38_39del AC	eliminating part hexanucleotide loop	1/1	Canada	[11]

**c.-161delC**	**+39delC**	**eliminating part hexanucleotide loop**	**1/2**	**Spain**	**[7]**
**c.-158-143del16**	**+42_57del16**	**eliminating IRE**	**1/3**	**France**	[11] **[73]**
c.-158-143del16	+42_57del16	eliminating IRE	NA	NA	[4]

Supplementary references

1. Castiglioni E, Soriani N, Girelli D, Camaschella C, Spiga I, Della Porta MG, Ferrari M, Cremonesi L: **High resolution melting for the identification of mutations in the iron responsive element of the ferritin light chain gene.** *Clin Chem Lab Med* 2010, **48:**1415-1418.
2. Cremonesi L, Fumagalli A, Soriani N, Ferrari M, Levi S, Belloli S, Ruggeri G, Arosio P: **Double-gradient denaturing gradient gel electrophoresis assay for identification of L-ferritin iron-responsive element mutations responsible for hereditary hyperferritinemia-cataract syndrome: identification of the new mutation C14G.** *Clin Chem* 2001, **47:**491-497.
3. Cazzola M, Bergamaschi G, Tonon L, Arbustini E, Grasso M, Vercesi E, Barosi G, Bianchi PE, Cairo G, Arosio P: **Hereditary hyperferritinemia-cataract syndrome: relationship between phenotypes and specific mutations in the iron-responsive element of ferritin light-chain mRNA.** *Blood* 1997, **90:**814-821.
4. Giansily M, Beaumont C, Desveaux C, Hetet G, Schved JF, Aguilar-Martinez P: **Denaturing gradient gel electrophoresis screening for mutations in the hereditary hyperferritinaemia cataract syndrome.** *Br J Haematol* 2001, **112:**51-54.
5. Rufer A, Howell JP, Lange AP, Yamamoto R, Heuscher J, Gregor M, Wuillemin WA: **Hereditary hyperferritinemia-cataract syndrome (HHCS) presenting with iron deficiency anemia associated with a new mutation in the iron responsive element of the L ferritin gene in a Swiss family.** *Eur J Haematol* 2011, **87:**274-278.
6. Bosio S, Campanella A, Gramaglia E, Porporato P, Longo F, Cremonesi L, Levi S, Camaschella C: **C29G in the iron-responsive element of L-ferritin: a new mutation associated with hyperferritinemia-cataract.** *Blood Cells Mol Dis* 2004, **33:**31-34.
7. Munoz-Munoz J, Cuadrado-Grande N, Moreno-Carralero MI, Hoyos-Sanabria B, Manubes-Guarch A, Gonzalez AF, Tejada-Palacios P, Del-Castillo-Rueda A, Moran-Jimenez MJ: **Hereditary hyperferritinemia cataract syndrome in four patients with mutations in the IRE of the FTL gene.** *Clin Genet* 2012.
8. Cicilano M, Zecchina G, Roetto A, Bosio S, Infelise V, Stefani S, Mazza U, Camaschella C: **Recurrent mutations in the iron regulatory element of L-ferritin in hereditary hyperferritinemia-cataract syndrome.** *Haematologica* 1999, **84:**489-492.
9. Volkmann M, Schiff JH, Hor M, Hentze MW, Fiehn W, Merkt J: **[Pair of siblings of Italian ethnicity with hyperferritinemia and cataract].** *Internist (Berl)* 2000, **41:**381-384.
10. Simsek S, Nanayakkara PW, Keek JM, Faber LM, Bruin KF, Pals G: **Two Dutch families with hereditary hyperferritinaemia-cataract syndrome and heterozygosity for an HFE-related haemochromatosis gene mutation.** *Neth J Med* 2003, **61:**291-295.
11. Hetet G, Devaux I, Soufir N, Grandchamp B, Beaumont C: **Molecular analyses of patients with hyperferritinemia and normal serum iron values reveal both L ferritin IRE and 3 new ferroportin (slc11A3) mutations.** *Blood* 2003, **102:**1904-1910.
12. van der Klooster JM: **[Hereditary hyperferritinaemia-cataract syndrome].** *Ned Tijdschr Geneeskd* 2003, **147:**1923-1928.
13. Cremonesi L, Paroni R, Foglieni B, Galbiati S, Fermo I, Soriani N, Belloli S, Ruggeri G, Biasiotto G, Cazzola M, et al: **Scanning mutations of the 5'UTR regulatory sequence of L-ferritin by denaturing high-performance liquid chromatography: identification of new mutations.** *Br J Haematol* 2003, **121:**173-179.
14. Vanita V, Hejtmancik JF, Hennies HC, Guleria K, Nurnberg P, Singh D, Sperling K, Singh JR: **Sutural cataract associated with a mutation in the ferritin light chain gene (FTL) in a family of Indian origin.** *Mol Vis* 2006, **12:**93-99.
15. Andonegui J, Prat M, Vargas M: **[Hereditary syndrome of hyperferritinemia and cataract].** *An Sist Sanit Navar* 2007, **30:**281-285.
16. Millonig G, Holzer MP, Tolle G, Auffarth GU, Muckenthaler MU, Seitz HK, Mueller S: **[Hereditary hyperferritinemia cataract syndrome--the first family in Germany].** *Z Gastroenterol* 2009, **47:**1211.

17. Shekunov J, de Groen PC, Lindor NM, Klee GG, Aleff RA, Wieben ED, Mohney BG: **Hereditary hyperferritinemia-cataract syndrome in two large multigenerational American families.** *J AAPOS* 2011, **15:**356-361.
18. Kato GJ, Casella F: **"L-ferritin Baltimore-1: A novel mutation in the iron responsive element (C32G) as cause of hyperferritinemia-cataract syndrome".** *Blood* 1999, **94:**407a.
19. Campagnoli MF, Pimazzoni R, Bosio S, Zecchina G, DeGobbi M, Bosso P, Oldani B, Ramenghi U: **Onset of cataract in early infancy associated with a 32G-->C transition in the iron responsive element of L-ferritin.** *Eur J Pediatr* 2002, **161:**499-502.
20. Craig JE, Clark JB, McLeod JL, Kirkland MA, Grant G, Elder JE, Toohey MG, Kowal L, Savoia HF, Chen C, et al: **Hereditary hyperferritinemia-cataract syndrome: prevalence, lens morphology, spectrum of mutations, and clinical presentations.** *Arch Ophthalmol* 2003, **121:**1753-1761.
21. Lachlan KL, Temple IK, Mumford AD: **Clinical features and molecular analysis of seven British kindreds with hereditary hyperferritinaemia cataract syndrome.** *Eur J Hum Genet* 2004, **12:**790-796.
22. Ismail AR, Lachlan KL, Mumford AD, Temple IK, Hodgkins PR: **Hereditary hyperferritinemia cataract syndrome: ocular, genetic, and biochemical findings.** *Eur J Ophthalmol* 2006, **16:**153-160.
23. Kroger A, Bachli EB, Mumford A, Gubler C: **Hyperferritinemia without iron overload in patients with bilateral cataracts: a case series.** *J Med Case Reports* 2011, **5:**471.
24. Wussuki-Lior O, Abu-Horowitz A, Netzer I, Almer Z, Morad Y, Goldich Y, Yahalom V, Pras E: **Hematologic biomarkers in childhood cataracts.** *Mol Vis* 2011, **17:**1011-1015.
25. Martin ME, Fargion S, Brissot P, Pellat B, Beaumont C: **A point mutation in the bulge of the iron-responsive element of the L ferritin gene in two families with the hereditary hyperferritinemia-cataract syndrome.** *Blood* 1998, **91:**319-323.
26. McLeod JL, Craig J, Gumley S, Roberts S, Kirkland MA: **Mutation spectrum in Australian pedigrees with hereditary hyperferritinaemia-cataract syndrome reveals novel and de novo mutations.** *Br J Haematol* 2002, **118:**1179-1182.
27. Sanders SJ, Suri M, Ross I: **Hereditary hyperferritinaemia-cataract syndrome and differential diagnosis of hereditary haemochromatosis.** *Postgrad Med J* 2003, **79:**600-601.
28. Wong K, Barbin Y, Chakrabarti S, Adams P: **A point mutation in the iron-responsive element of the L-ferritin in a family with hereditary hyperferritinemia cataract syndrome.** *Can J Gastroenterol* 2005, **19:**253-255.
29. Assia N, Goldenberg-Cohen N, Rechavi G, Amariglio N, Cohen Y: **Mutation analysis of the ferritin L-chain gene in age-related cataract.** *Mol Vis* 2010, **16:**2487-2493.
30. Holzgreve H: **[Hereditary hyperferritinemia cataract syndrome].** *Dtsch Med Wochenschr* 2011, **136:**1895-1898.
31. Durupt S, Durieu I, Salles B, Beaumont C, Hot A, Rousset H, Vital Durand D: **[A potential etiology of elevated ferritin: hyperferritinemia-cataract syndrome].** *Rev Med Interne* 2001, **22:**83-85.
32. Balas A, Aviles MJ, Garcia-Sanchez F, Vicario JL: **Description of a new mutation in the L-ferrin iron-responsive element associated with hereditary hyperferritinemia-cataract syndrome in a Spanish family.** *Blood* 1999, **93:**4020-4021.
33. Brooks DG, Manova-Todorova K, Farmer J, Lobmayr L, Wilson RB, Eagle RC, Jr., St Pierre TG, Stambolian D: **Ferritin crystal cataracts in hereditary hyperferritinemia cataract syndrome.** *Invest Ophthalmol Vis Sci* 2002, **43:**1121-1126.
34. Ladero JM, Balas A, Garcia-Sanchez F, Vicario JL, Diaz-Rubio M: **Hereditary hyperferritinemia-cataract syndrome. Study of a new family in Spain.** *Rev Esp Enferm Dig* 2004, **96:**507-509, 510-501.
35. Garcia Erce JA, Cortes T, Cremonesi L, Cazzola M, Perez-Lungmus G, Giralt M: **[Hyperferritinemia-cataract syndrome associated to the HFE gene mutation. Two new Spanish families and a new mutation (A37T: "Zaragoza")].** *Med Clin (Barc)* 2006, **127:**55-58.
36. Cao W, McMahon M, Wang B, O'Connor R, Clarkson M: **A case report of spontaneous mutation (C33>U) in the iron-responsive element of L-ferritin causing hyperferritinemia-cataract syndrome.** *Blood Cells Mol Dis* 2010, **44:**22-27.

37. Alvarez-Coca-Gonzalez J, Moreno-Carralero MI, Martinez-Perez J, Mendez M, Garcia-Ros M, Moran-Jimenez MJ: **The hereditary hyperferritinemia-cataract syndrome: a family study.** *Eur J Pediatr* 2010, **169:**1553-1555.
38. Del Castillo-Rueda A, Moreno-Carralero MI, Cuadrado-Grande N, Mendez M, Moran-Jimenez MJ: **[Hyperferritinemia, ferropenia and metabolic syndrome in a patient with a new mutation of gene TFR2 and another in gene FTL. A family study].** *Med Clin (Barc)* 2011, **137:**68-72.
39. Nonnenmacher L, Langer T, Blessing H, Gabriel H, Buchwald HJ, Meneksedag C, Kohne E, Gencik M, Debatin KM, Cario H: **Hereditary hyperferritinemia cataract syndrome: clinical, genetic, and laboratory findings in 5 families.** *Klin Padiatr* 2011, **223:**346-351.
40. Mumford AD, Vulliamy T, Lindsay J, Watson A: **Hereditary hyperferritinemia-cataract syndrome: two novel mutations in the L-ferritin iron-responsive element.** *Blood* 1998, **91:**367-368.
41. Bertola F, Veneri D, Bosio S, Battaglia P, Disperati A, Schiavon R: **Hyperferritinaemia without iron overload: pathogenic and therapeutic implications.** *Curr Drug Targets Immune Endocr Metabol Disord* 2004, **4:**93-105.
42. Ferrari F, Foglieni B, Arosio P, Camaschella C, Daraio F, Levi S, Garcia Erce JA, Beaumont C, Cazzola M, Ferrari M, Cremonesi L: **Microelectronic DNA chip for hereditary hyperferritinemia cataract syndrome, a model for large-scale analysis of disorders of iron metabolism.** *Hum Mutat* 2006, **27:**201-208.
43. Serra M, Longo F, Roetto A, Sandri A, Piga A: **A child with hyperferritinemia: case report.** *Ital J Pediatr* 2011, **37:**20.
44. Garderet L, Hermelin B, Gorin NC, Rosmorduc O: **Hereditary hyperferritinemia-cataract syndrome: a novel mutation in the iron-responsive element of the L-ferritin gene in a French family.** *Am J Med* 2004, **117:**138-139.
45. Papanikolaou G, Chandrinou H, Bouzas E, Contopoulos-Ioannidis D, Kalotychou V, Prentzas K, Lilakos K, Asproudis I, Palaiologou D, Premetis E, et al: **Hereditary hyperferritinemia cataract syndrome in three unrelated families of western Greek origin caused by the C39 > G mutation of L-ferritin IRE.** *Blood Cells Mol Dis* 2006, **36:**33-40.
46. Arosio C, Fossati L, Vigano M, Trombini P, Cazzaniga G, Piperno A: **Hereditary hyperferritinemia cataract syndrome: a de novo mutation in the iron responsive element of the L-ferritin gene.** *Haematologica* 1999, **84:**560-561.
47. Fritsche-Polanz R, Wallner M, Cohen G, Eberle C, Sunder-Plassmann G, Fodinger M: **Granulocyte function in patients with L-ferritin iron-responsive element (IRE) 39C-->T-positive hereditary hyperferritinaemia-cataract syndrome.** *Eur J Clin Invest* 2004, **34:**701-708.
48. Ferrante M, Geubel AP, Fevery J, Marogy G, Horsmans Y, Nevens F: **Hereditary hyperferritinaemia-cataract syndrome: a challenging diagnosis for the hepatogastroenterologist.** *Eur J Gastroenterol Hepatol* 2005, **17:**1247-1253.
49. Hernandez Martin D, Cervera Bravo A, Balas Perez A: **[Hereditary hyperferritinemia and cataract syndrome: a de novo mutation].** *An Pediatr (Barc)* 2008, **68:**408-410.
50. Messa E, Pellegrino RM, Palmieri A, Carturan S, Cilloni D, Saglio G, Roetto A: **Identification of a novel mutation in the L ferritin iron-responsive element causing hereditary hyperferritinemia-cataract syndrome.** *Acta Haematol* 2009, **122:**223-225.
51. Perez-Lucena MJ, Moreno-Rosel MS, Sagarra-Tió M, Félez J: **On the Hyperferritinemia and Hereditary Cataract Syndrome.** *Open J Blood Dis* 2012, **2:**11-13.
52. Beaumont C, Leneuve P, Devaux I, Scoazec JY, Berthier M, Loiseau MN, Grandchamp B, Bonneau D: **Mutation in the iron responsive element of the L ferritin mRNA in a family with dominant hyperferritinaemia and cataract.** *Nat Genet* 1995, **11:**444-446.
53. Aguilar-Martinez P, Biron C, Masmejean C, Jeanjean P, Schved JF: **A novel mutation in the iron responsive element of ferritin L-subunit gene as a cause for hereditary hyperferritinemia-cataract syndrome.** *Blood* 1996, **88:**1895.
54. Arnold JD, Mumford AD, Lindsay JO, Hegde U, Hagan M, Hawkins JR: **Hyperferritinaemia in the absence of iron overload.** *Gut* 1997, **41:**408-410.
55. Barton JC, Beutler E, Gelbart T: **Coinheritance of alleles associated with hemochromatosis and hereditary hyperferritinemia-cataract syndrome.** *Blood* 1998, **92:**4480.

56. Mumford AD, Cree IA, Arnold JD, Hagan MC, Rixon KC, Harding JJ: **The lens in hereditary hyperferritinaemia cataract syndrome contains crystalline deposits of L-ferritin.** *Br J Ophthalmol* 2000, **84:**697-700.
57. Perez de Nanclares G, Castano L, Martul P, Rica I, Vela A, Sanjurjo P, Aldamiz-Echevarria K, Martinez R, Sarrionandia MJ: **Molecular analysis of hereditary hyperferritinemia-cataract syndrome in a large Basque family.** *J Pediatr Endocrinol Metab* 2001, **14:**295-300.
58. Mohn A, Capanna R, Chiarelli F: **A girl with persistent hyperferritinaemia.** *Lancet* 2005, **365:**1744.
59. Del Castillo Rueda A, Fernandez Ruano ML: **[Hereditary hyperferritinemia cataracts syndrome in a Spanish family caused by the A40G mutation (Paris) in the L-ferritin (FTL) gene associated with the mutation H63D in the HFE gene].** *Med Clin (Barc)* 2007, **129:**414-417.
60. Hughes M, Vosylius P: **Dual diagnoses of hereditary hyperferritinaemia-cataract syndrome and hereditary haemochromatosis.** *Clin Lab Haematol* 2006, **28:**357-359.
61. Rochow N, Bachmaier N, Tost F, Beck JF, Bernig T: **The case of a 1-year-old girl with hereditary hyperferritinemia cataract syndrome.** *Pediatr Hematol Oncol* 2009, **26:**136-141.
62. Arnold J, Sangwaiya A, Manglam V, Thursz M, Beaumont C, Kannengiesser C, Busbridge M: **Hepcidin levels in hereditary hyperferritinemia: Insights into the iron-sensing mechanism in hepatocytes.** *World J Gastroenterol* 2010, **16:**3541-3545.
63. Platteau E, Hoornaert K, Moens K, Leroy BP: **Age-dependent ocular phenotype in hereditary hyperferritinaemia cataract syndrome (HHCS).** *Acta Ophthalmol* 2012, **90:**e239-240.
64. Girelli D, Corrocher R, Bisceglia L, Olivieri O, De Franceschi L, Zelante L, Gasparini P: **Molecular basis for the recently described hereditary hyperferritinemia-cataract syndrome: a mutation in the iron-responsive element of ferritin L-subunit gene (the "Verona mutation").** *Blood* 1995, **86:**4050-4053.
65. Meneses FG, Schnabel B, Silva ID, Alberto FL, Toma L, Nader HB, Lopes CC: **Identification of the mutations associated with hereditary hyperferritinemia cataract syndrome and hemochromatosis in a Brazilian family.** *Clin Genet* 2011, **79:**189-192.
66. Phillips JD, Warby CA, Kushner JP: **Identification of a novel mutation in the L-ferritin IRE leading to hereditary hyperferritinemia-cataract syndrome.** *Am J Med Genet A* 2005, **134A:**77-79.
67. Gonzalez-Huerta L, Ramirez-Sanchez V, Rivera-Vega M, Messina-Baas O, Cuevas-Covarrubias S: **A family with hereditary hyperferritinaemia cataract syndrome: evidence of incomplete penetrance and clinical heterogeneity.** *Br J Haematol* 2008, **143:**596-598.
68. Camaschella C, Zecchina G, Lockitch G, Roetto A, Campanella A, Arosio P, Levi S: **A new mutation (G51C) in the iron-responsive element (IRE) of L-ferritin associated with hyperferritinaemia-cataract syndrome decreases the binding affinity of the mutated IRE for iron-regulatory proteins.** *Br J Haematol* 2000, **108:**480-482.
69. Chang-Godinich A, Ades S, Schenkein D, Brooks D, Stambolian D, Raizman MB: **Lens changes in hereditary hyperferritinemia-cataract syndrome.** *Am J Ophthalmol* 2001, **132:**786-788.
70. Burdon KP, Sharma S, Chen CS, Dimasi DP, Mackey DA, Craig JE: **A novel deletion in the FTL gene causes hereditary hyperferritinemia cataract syndrome (HHCS) by alteration of the transcription start site.** *Hum Mutat* 2007, **28:**742.
71. Girelli D, Corrocher R, Bisceglia L, Olivieri O, Zelante L, Panozzo G, Gasparini P: **Hereditary hyperferritinemia-cataract syndrome caused by a 29-base pair deletion in the iron responsive element of ferritin L-subunit gene.** *Blood* 1997, **90:**2084-2088.
72. Cazzola M, Foglieni B, Bergamaschi G, Levi S, Lazzarino M, Arosio P: **A novel deletion of the L-ferritin iron-responsive element responsible for severe hereditary hyperferritinaemia-cataract syndrome.** *Br J Haematol* 2002, **116:**667-670.
73. Feys J, Nodarian M, Aygalenq P, Cattan D, Bouccara AS, Beaumont C: **[Hereditary hyperferritinemia syndrome and cataract].** *J Fr Ophtalmol* 2001, **24:**847-850.
